# Comprehensive application of AI algorithms with TCR NGS data for glioma diagnosis

**DOI:** 10.1038/s41598-024-65305-9

**Published:** 2024-07-04

**Authors:** Kaiyue Zhou, Zhengliang Xiao, Qi Liu, Xu Wang, Jiaxin Huo, Xiaoqi Wu, Xiaoxiao Zhao, Xiaohan Feng, Baoyi Fu, Pengfei Xu, Yunyun Deng, Wenwen Xiao, Tao Sun, Lin Da

**Affiliations:** 1 Department of Mathematics, School of Mathematical Sciences, Inner Mongolia University, Hohhot, China; 2 Hangzhou ImmuQuad Biotechnologies, LLC, Hangzhou, China; 3 Institute of Wenzhou, Zhejiang University, Wenzhou, China

**Keywords:** Glioma, T-cell repertoire (TCR), Prognosis, Artificial intelligence techniques, Feature selection methods, CNS cancer, Cancer genomics, Tumour biomarkers, Cancer genomics, CNS cancer, Tumour biomarkers

## Abstract

T-cell receptor (TCR) detection can examine the extent of T-cell immune responses. Therefore, the article analyzed characteristic data of glioma obtained by DNA-based TCR high-throughput sequencing, to predict the disease with fewer biomarkers and higher accuracy. We downloaded data online and obtained six TCR-related diversity indices to establish a multidimensional classification system. By comparing actual presence of the 602 correlated sequences, we obtained two-dimensional and multidimensional datasets. Multiple classification methods were utilized for both datasets with the classification accuracy of multidimensional data slightly less to two-dimensional datasets. This study reduced the TCR β sequences through feature selection methods like RFECV (Recursive Feature Elimination with Cross-Validation). Consequently, using only the presence of these three sequences, the classification AUC value of 96.67% can be achieved. The combination of the three correlated TCR clones obtained at a source data threshold of 0.1 is: CASSLGGNTEAFF_TRBV12_TRBJ1-1, CASSYSDTGELFF_TRBV6_TRBJ2-2, and CASSLTGNTEAFF_TRBV12_TRBJ1-1. At 0.001, the combination is: CASSLGETQYF_TRBV12_TRBJ2-5, CASSLGGNQPQHF_TRBV12_TRBJ1-5, and CASSLSGNTIYF_TRBV12_TRBJ1-3. This method can serve as a potential diagnostic and therapeutic tool, facilitating diagnosis and treatment of glioma and other cancers.

## Introduction

In recent years, the application of AI algorithms in medicine has become more widespread and intensive, with researchers gradually applying the technology to challenging processes such as cancer diagnosis, treatment, and prognosis. Cancer data encompasses diverse modalities, including medical imaging for visual information and molecular data obtained from biopsies of solid tumors and liquid samples. As a result, numerous studies have focused on analyzing these different modalities using corresponding machine learning algorithms to advance cancer diagnosis, monitoring, and treatment^1^.

While imaging studies have greatly impacted current cancer diagnosis and treatment, the rapid progress in sample processing, genome sequencing, and molecular technologies has highlighted the importance of molecular data analysis in cancer research. Molecular data, particularly from solid tumor biopsies and molecular liquid biopsies, contain fine-grained features that are not available in imaging data. This has led to an increasing focus on molecular data analysis in cancer research^1^. For instance, the diagnosis of hematologic malignancies can be classified using various blood cell parameters extracted from Cell Population Data (CPD), combined with machine learning classification algorithms^2^. The study found that the artificial neural network (ANN) algorithm achieved the highest AUC (Area Under the Curve) of approximately 93.5%.

TCR NGS (Next-generation sequencing) data is a type of molecular data that holds significance in cancer research. NGS stands for Next-Generation Sequencing, which is currently the most widely used high-throughput DNA sequencing technology. NGS enables in-depth analysis of the immune repertoire. Research in the fields of T-cell receptors (TCR) and immunoglobulins (IG) helps in understanding immune-related diseases^3^. TCR, short for T-cell receptor, is a receptor found on the surface of T lymphocytes that plays a crucial role in recognizing and binding antigens, triggering and regulating the immune response. TCR chains consist of variable and constant regions, with the variable region containing complementarity determining regions (CDRs) responsible for TCR specificity and antigen recognition. The constant region provides structural support and signaling functions. The diversity and assortment of TCRs primarily stem from the highly diverse Complementarity Determining Region 3 (CDR3), which is formed through rearrangement and alteration of V(D)J segments. These V(D)J regions, found within the genes responsible for coding TCRs, contribute to the specificity and heterogeneity of TCRs. In the study by John-William Sidhom et al., the researchers modeled the highly complex TCR sequencing data using joint representation learning of CDR3 sequences and V/D/J genes. This approach provided improved TCR characterization across multiple human and mouse datasets, as well as extraction of antigen-specific TCRs from noisy single-cell RNA-Seq and T cell culture assays^4^.

Several studies suggest that TCR diversity plays a crucial role in the diagnosis and treatment of cancer. For instance, a study by Yang-Yang Liu and colleagues observed significant differences in the TCR repertoire between lung cancer patients and healthy individuals, including differences in CDR3 clonotype, diversity, V/J segment utilization, and sequence^5^. Additionally, Huaichao Luo et al. constructed a machine learning classification model for diagnosing pulmonary nodules based on features of peripheral blood T-cell receptor repertoire, such as the Shannon Index, combined with age and corresponding data on benign and malignant pulmonary nodules^6^. The model achieved an AUC value of 0.8, indicating that TCR diversity indices contain relevant information for pulmonary nodules or lung cancer. In this study, we plan to analyze the importance of the T-cell diversity indices to the distinction of glioma patients and healthy individuals.

In addition to TCR diversity indices, several studies have demonstrated the accurate use of TCR sequence distribution and presence data for disease diagnosis and analysis^7,8^. This forms the focus of our paper.

Gliomas, including glioblastoma, are tumors that originate from precursor or glial cells^9^. According to the CBTRUS (Central Brain Tumor Registry of the United States) Statistical Report, the incidence rate of glioblastoma is reported as high as 3.19 cases per 100,000, but the five-year survival rate for glioblastoma is significantly low at 4.67%. These statistics highlight the prevalence of glioblastoma and the need for effective diagnostic approaches and emphasize the urgency to improve early detection and accurate diagnosis of glioma^9^.

## Results

### Data acquisition and data management

The proposed data was divided into two groups: 44 training samples and 30 testing samples, with an equal split between positive and negative cases. The data of 22 positive glioma patients in the training set and 15 positive glioma patients in the test set were obtained from 2 researches^10,11^. Additionally, a total of 37 healthy human negative samples from the training and test samples were obtained from IMMUNOSEQ ANALYZER (https://clients.adaptivebiotech.com/).

The raw data from the training samples underwent high-throughput TCR sequencing analysis, resulting in a total of 1,527,623 TCR β sequences. We obtained information about the presence of each sequence in the samples. However, due to the enormous size of the data, it is challenging to incorporate it directly into the classification model. At the same time, cluster identification in high-dimensional sequencing data also faces challenges due to its inherent complexity^12^. Therefore, we performed feature selection using Fisher's exact test as we described previously^8^. We screened 602 TCR β sequences with a P-value less than 0.1, which exhibited a higher correlation with glioma diagnosis, to be used as features for determining glioma.

### Classification effects on TCR diversity indices

For these 74 samples, we analyzed TCR diversity, clonality and singleton frequency. According to the common bioindicator system, the article obtains each common immunome sequencing result index corresponding to positive negative samples, such as Clonality index, Shannon diversity index, inverse Simpson diversity index, VJ diversity index, Singleton ratio, and Simpson diversity index these 6 indices.

Of the above indices, the Clonality index is used to measure the extent of TCR clone amplification and to assess the number and proportion of major clones in tumors, with higher values indicating a higher frequency of amplification of some TCR clones. Clonality takes values between 0 and 1, with a value of 0 indicating that each individual is unique and no clone is amplified; a value of 1 indicating that all individuals are copies of the same clone. The index is calculated as follows, where productive unique represents the total number of TCR clones in the sample and diversity is the result of the Shannon diversity index^13^.

$$ {\text{Clonality}} = 1 - \frac{{{\text{diversity}}}}{{\ln \;({\text{productive}}\;{\text{uniques}})}} $$

The Hvj diversity index is used to measure the diversity of V-J gene combinations of TCR clones in the sample. The index measures the frequency of the ith V-J gene fragment combination use type in the sample by the ratio of pi, thus reflecting the degree of T cell expansion. This index can also be used to reflect the replicative proliferation capacity following stress generated by T-specific recognition of antigen, with higher values indicating a greater degree of clonal amplification, which is calculated as follows^14^.

$$ H^{\prime} = - { }\sum {\text{p}}_{{\text{i}}} {\text{ln}}\left( {{\text{p}}_{{\text{i}}} } \right) $$

where p_i_ stands for the frequency of the ith V-J gene fragment combination.

The Shannon Index of diversity is used to measure the diversity of TCR clones in the sample. This index incorporates information regarding species richness (the number of different clone types present) and the evenness of the distribution of individuals among these clone types. A higher Shannon index signifies a greater diversity of TCR clones in the sample. The pi in the formula represents the frequency of the ith specific clone type. Thus, the Shannon index considers the number of TCR clone types and the frequency evenness of each clone^15^.

$$ H^{\prime} = - { }\sum {\text{p}}_{{\text{i}}} {\text{ln}}\left( {{\text{p}}_{{\text{i}}} } \right) $$

where p_i_ here stands for the frequency of the frequency of the ith specific clone type.

The Singleton Frequency is used to represent the proportion of Naïve T cells frequency in a sample and is a metric used to assess the degree of backbone of immune system^16^. In TCR studies, several studies have shown that the number of singletons is positively correlated with the body's ability to resist unfamiliar diseases, so this indicator can also reflect the degree of the body's immune response to the disease. The calculation formula is as follows, where n_1 indicates the number of clone types that appear only once in the sample and n indicates the number of all clone types in the sample.

$$ {\text{Singleton }} = {\text{ n}}\_1/({\text{n}} - 1) $$

**Figure 1:**
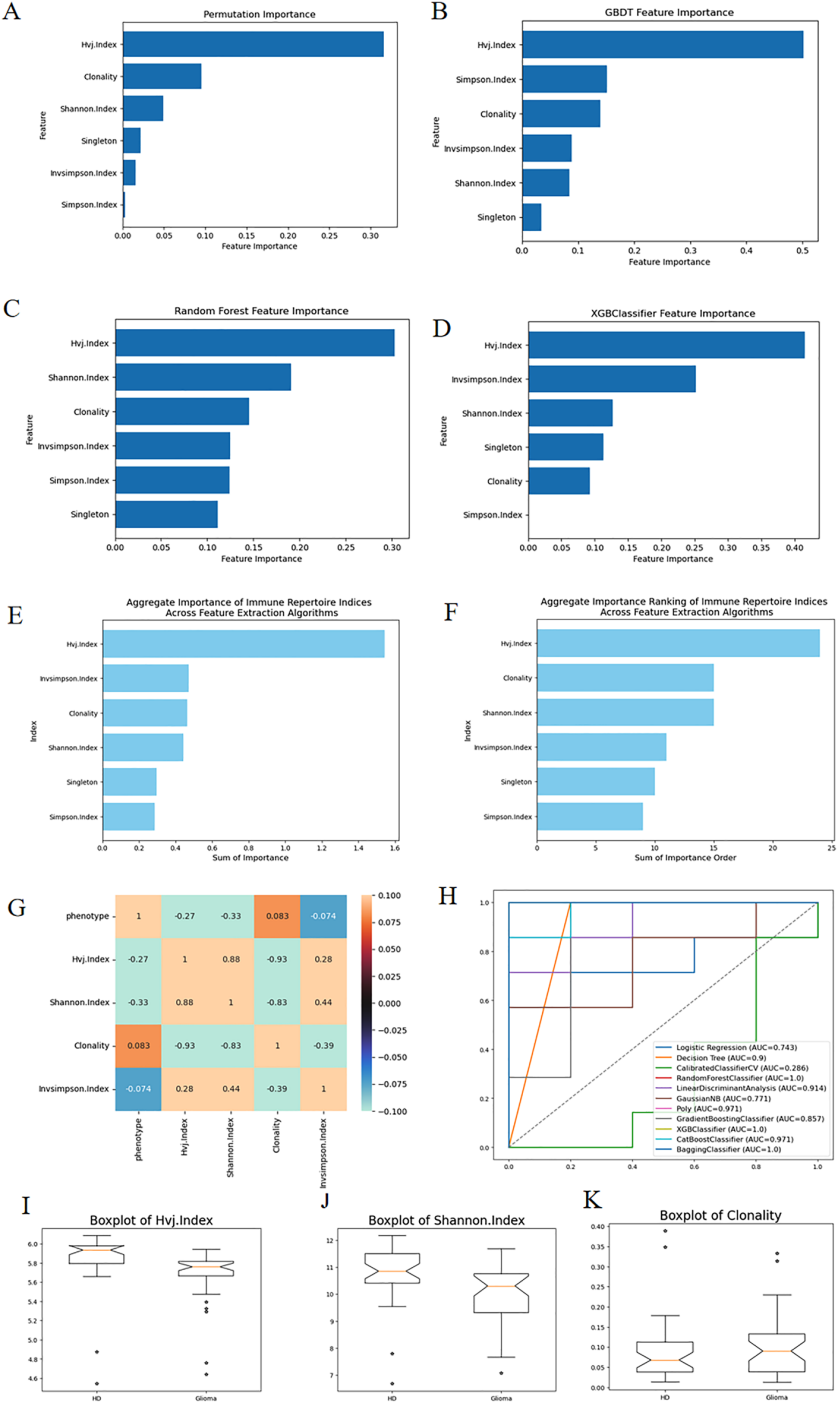
(**A**–**D**) show the results of the four feature selection methods. (**E**,**F**) are the results of feature selection process of RFECV (Recursive Feature Elimination with Cross-Validation) algorithms, representing the sum of importance and the sum of the ordering of importance respectively. (**G**) is the correlation coefficient matrix of phenotype and three relevant indicators and one irrelevant indicator. With these three associated diversity indicators, we can attain good classification AUC as is shown in the ROC curves in (**H**). (**I**–**K**) are three box line plots of the three indicators among HD and glioma.

To assess the importance of six indicators, we employed four feature selection methods such as GBDT (Gradient Boosting Decision Tree), RF (Random Forest), XGBoost (Extreme Gradient Boosting), Permutation to rank the importance of this dataset. The corresponding bar graphs are illustrated in Fig. 1A–D. Figure 1E,F represent the sum of importance and sum of importance rank derived from the four feature selection methods respectively. It is evident that the VJ diversity index (Hvj.Index) holds the highest importance, followed by the proportion of monoclonal T cells (Singleton) and Simpson's diversity index (Invsimpson.Index). Subsequently, Fig. 1I–K display box plots for these three indicators corresponding to positive–negative samples, confirming their distinct differences.

Furthermore, Fig. 1G showcases a correlation coefficient matrix between various indicators and phenotypes. It reveals that the three indicators with higher importance exhibit stronger correlations with phenotypes. Conversely, the indicator with the lowest importance, the Clonality index, demonstrates the weakest correlation with phenotypes.

Figure 1H illustrates the comprehensive ROC (Receiver Operating Characteristic) curves derived from 11 classification algorithms based on the datasets of TCR Diversity Indices and phenotype of the samples. It can be observed that the Random Forest, XGBoost, and Bagging classifiers all achieve a 100% classification AUC value. The three box plots among HD (Health Donor) and Glioma patients in Fig. 1I–K provide further evidence supporting the aforementioned statement.

### Binary classification algorithms on TCR sequences

According to our previous two-dimensional classification paper, it has been demonstrated that extracting two-dimensional features from high-throughput sequencing results can improve diagnostic outcomes^8^. In the context of glioma, the two-dimensional data features extracted from high-throughput sequencing results correspond to a specific threshold of Fisher's exact test P-value. These features include the total clonetypes and associated clonetypes. The total clonetypes represent the total number of distinct TCRβ sequence species observed in each sample, while the associated clonetypes denote the number of repeated species with associated TCR sequences. With the two-dimensional data above, we built up the classification system with 16 algorithms including neural network algorithms, ensemble learning algorithms and traditional machine learning algorithms. The exact classification result of each algorithm is presented in the Supplementary Table 1.

**Figure 2:**
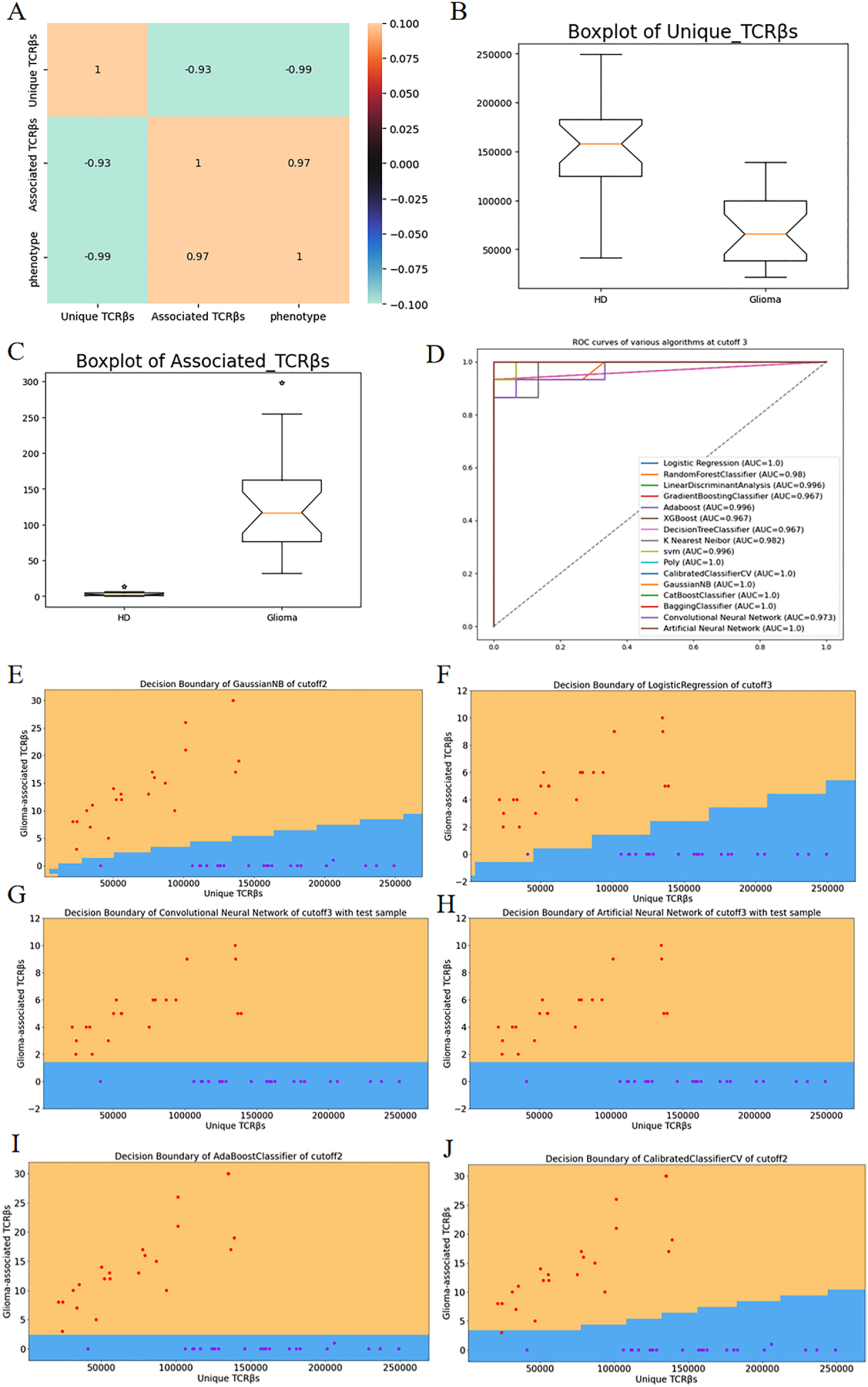
(**A**) displays correlation coefficient plots illustrating the relationship between the two-dimensional features and phenotype factors. (**B**,**C**) present Box plots showcasing the unique TCRβs and associated TCRβs, respectively, both of which exhibit high relevance to the phenotype. (**D**) showcases ROC curves for the two-dimensional datasets with a P-value cutoff of 0.001, demonstrating high AUC values. (**E**–**J**) shows the decision boundaries of various algorithms at different P-value cutoffs.

In Fig. 2A, the correlation coefficient plots illustrate the strong correlation between the two-dimensional features and phenotype factors. Specifically, the number of associated clonetypes shows significant relevance to the phenotype, particularly in relation to glioma. This is further confirmed by the box graphs, which reveal a higher number of associated clonetypes in glioma patients compared to healthy individuals. Based on these findings, we construct classifiers using the two-dimensional extracted features from each sample. The data is analyzed using 8 classical classifiers, 4 ensemble learning algorithms, and 2 neural network algorithms. The resulting ROC curves are combined in Fig. 2D, demonstrating that these algorithms achieve excellent classification performance in the two-dimensional data. Some algorithms even achieve an AUC of 1, indicating perfect classification. This highlights the effectiveness of the two-dimensional feature extraction method for diagnosing glioma. In Fig. 2E–J, we present the decision boundaries corresponding to several algorithms that exhibit good classification results.

### Multidimensional classification algorithms on TCR sequences

The multidimensional dataset of TCR β sequences uses 0–1 format data to represent the presence of each sequence in each sample. If the data equals 0, the sequence is absent in the subject, else the sequence exists in the sample. Multidimensional classification algorithms were applied to analyze the tabular data frames of glioma, which contained information on the presence or absence of relevant TCRβs. Several classification algorithms were used, including random forest, support vector machine with linear and polynomial kernel functions, logistic regression, linear discriminant analysis, Bayesian algorithm, GBDT, XGB, and decision tree algorithm.

If the data used to train machine learning models varies in quality, the predicted results may be inaccurate or misleading^17^. To identify marker sequences specific to glioma while using fewer markers and maintaining high accuracy, feature selection techniques were employed. Fisher's exact test, a commonly used method in bioinformatics, was utilized for feature selection. By applying different cutoffs for Fisher's exact test, groups of sequences with varying degrees of relevance to gliomas were identified. These sequences were then incorporated into the classification algorithm system to achieve higher classification accuracy using a reduced number of sequences. This approach allows for the identification of marker sequences specifically associated with glioma. The exact classification result of each algorithm is presented in the Supplementary Table 2.

**Figure 3:**
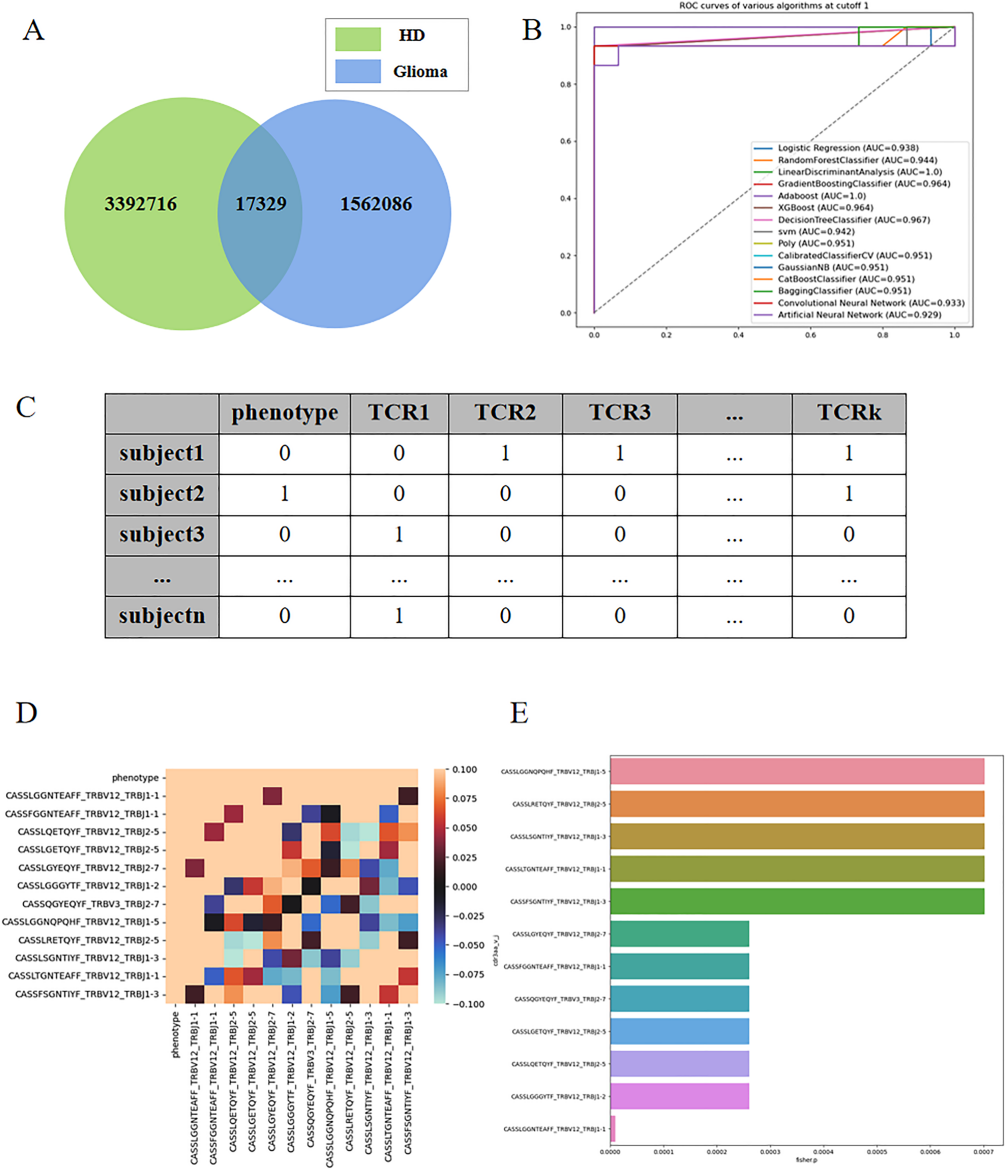
(**A**) presents a Venn diagram illustrating the intersection of sequence species between the healthy and glioma patient groups. (**B**) shows the AUC (Area Under the Curve) obtained from 602 sequences with a cutoff value of 0.001. (**C**) displays a tabular dataset with multidimensional columns, where each column represents 0–1 data indicating the presence or absence of TCR β sequences in the samples. A value of 1 signifies the presence of the sequence, while 0 indicates its absence. (**D**) presents correlation coefficient graphs corresponding to the multidimensional datasets at a P-value cutoff of 0.001. (**E**) is a bar chart illustrating the importance of the TCR β sequences, with the values corresponding to the P-values of 0.001.

Figure 3A presents a Venn diagram depicting the intersection of sequence species between the healthy and glioma patient groups. It is evident that the sequences in the two groups exhibit high divergence, with only a small fraction being common to both.

The linear discriminant method achieved the highest accuracy, with an AUC of 100% (Fig. 3B: AUC obtained from 602 sequences with cutoff1 data). In this study, we obtained P-values using Fisher's exact test and selected TCRβ sequences with P-values less than or equal to 10^−2^, 10^−3^, and 10^−4^, resulting in 39, 12 sequences, and 1 relevant sequence, respectively.

As depicted in Fig. 3C, each column represents binary data (0 or 1) indicating the presence or absence of specific TCRβ sequences in the corresponding samples. We hypothesized that the presence or absence of these sequences is closely associated with glioma status. By analyzing the sequences, we identified a set of TCRβ sequences directly related to glioma and utilized a small number of sequences to accurately predict the disease status.

Next, we applied these sets of sequence data to the 12 classification algorithms mentioned earlier. Notably, when selecting a P-value of 10^−3^, several classification algorithms also achieved an AUC of 1. Consequently, we narrowed down the factors to these 12 sequence factors. Figure 3D displays the correlation coefficient graphs, indicating that the phenotypes exhibit a relatively high correlation with these sequences, while the correlation with quality control is generally lower.

By utilizing only these 12 TCRβ sequences, we achieved a high prediction accuracy rate. The specific sequences are as follows: CASSLGGNQPQHF_TRBV12_TRBJ1-5, CASSLRETQYF_TRBV12_TRBJ2-5, CASLSGNTIYF_TRBV12_TRBJ1-3, CASLTGNTEAFF_TRBV12_TRBJ1-1, CASSFSGNTIYF_TRBV12_TRBJ1-3, CASSLGYEQYF_TRBV12_TRBJ2-7, CASFGGNTEAFF_TRBV12_TRBJ1-1, CASQGYEQYF_TRBV3_TRBJ2-7, CASLGETQYF_TRBV12_TRBJ2-5, CASSLQETQYF_TRBV12_TRBJ2-5, CASLGGGYTF_TRBV12_TRBJ1-2, CASLGGNTEAFF_TRBV12_TRBJ1-1. Figure 3E presents a bar chart illustrating the importance corresponding to the P-values of these 12 sequences.

### Core features’ selection on TCR sequences

Due to computational limitations, we did not perform differential gene analysis on the initial millions of sequences. Instead, we focused on 602 sequences corresponding to Fisher's exact test threshold of 0.1. Based on this subset of sequences, we explored feature extraction methods that outperformed Fisher's exact test and identified TCRβ sequences that were more significant for glioma diagnosis. In many cases, the data only describes one chain, and when the α chain lacks data, it is mainly the β chain of TCR. This is a big defect in screening antigen-binding TCR, because the complementary sequence of α-and β-chains determines the specificity of TCR binding^18^.

Apart from Fisher's exact test, this study primarily utilized two algorithms for feature extraction: Lasso and RFECV. We employed the Lasso algorithm to select features from the multidimensional data obtained through Fisher's exact test using threshold values of 0.1, 0.01, and 0.001. In this study, RFECV was combined with nine classification algorithms for feature extraction, and the effectiveness of feature extraction was evaluated using 12 classification algorithms separately. This approach resulted in improved feature extraction, yielding higher classification accuracy with fewer sequences.

Although the two-dimensional features extracted from Fisher's exact test in Fig. 2 show promising results in distinguishing healthy individuals from glioma patients, we aim to explore additional approaches to identify disease-associated sequences for easier disease diagnosis and future drug development. In the following analysis, we focus on the screening of glioma-associated sequences.

**Figure 4:**
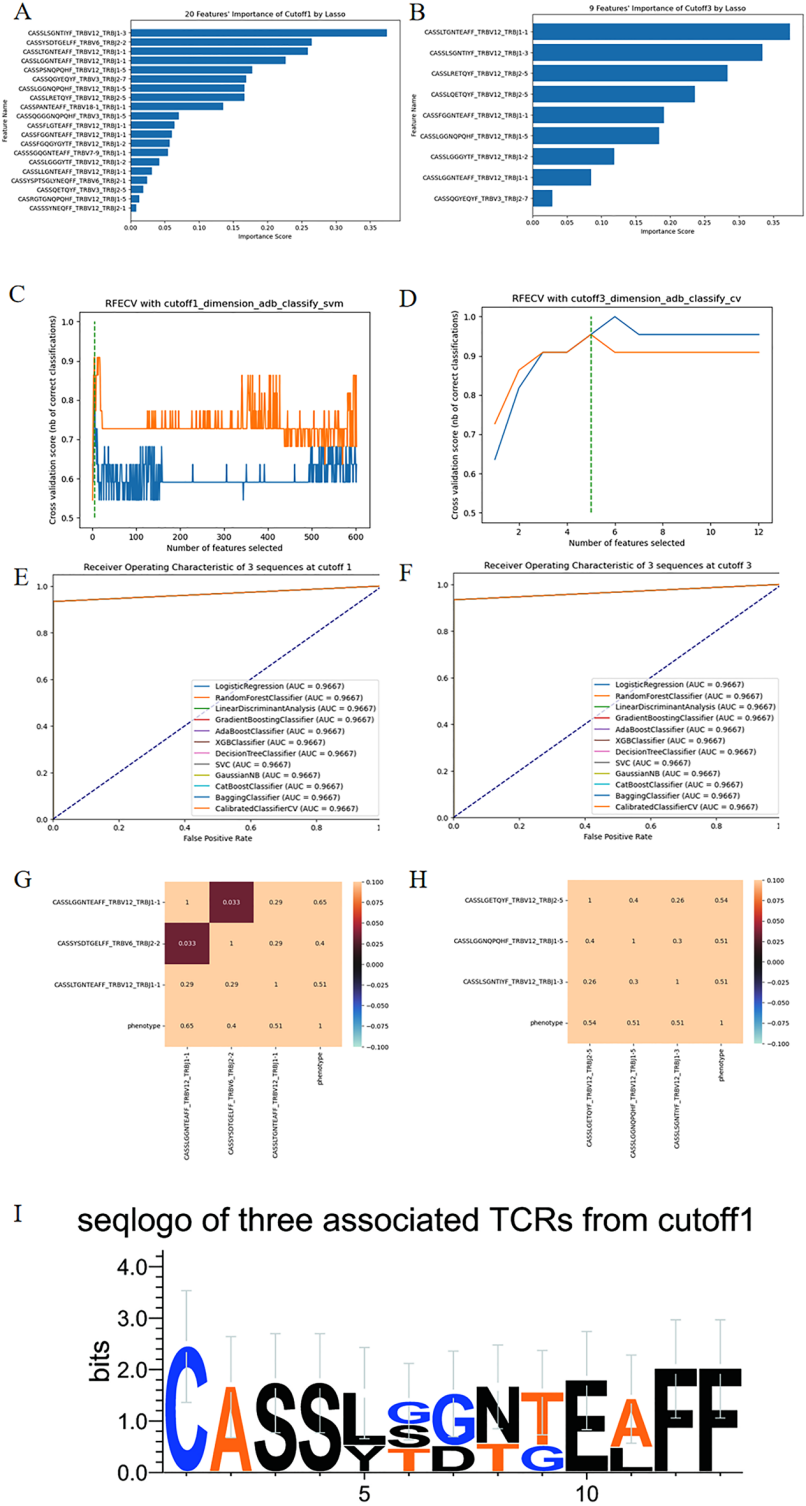
(**A**,**B**) represent the distribution of importance scores obtained from Lasso with threshold values of 0.1 and 0.001, respectively. (**C**,**D**) demonstrate the optimal performance of RFECV, indicating that 5 TCRβ sequences yield the highest AUC. (**E**,**F**) reveal the ROC curves of the two groups of core sequences and phenotype. (**G**,**H**) depicts the correlation coefficient plots of the two groups of core sequences. (**I**) shows the seqlogo image representing the core 3 TCRβ sequences obtained from the RFECV feature selection algorithm.

Figure 4A,B illustrate the importance distribution of Lasso with threshold values of 0.1 and 0.001, respectively. We determined that a threshold value of 0.1 yielded an appropriate selection of 19 dimensions for feature selection, while the thresholds of 0.01 and 0.001 resulted in 11 and 9 dimensions, respectively. There are still too many sequences in relative terms.

While when it comes to the method of RFECV algorithm, as is shown in the Fig. 4C, for the 602 sequences with a threshold of 0.1 for feature extraction, the feature extraction algorithm employed is Adaboost (Adaptive Boosting), and the classification algorithm used is SVM (Support Vector Machine). This approach yields a feature dimension of 5, achieving an AUC of 0.967 and an accuracy of 0.967. The five selected sequences are CASSLGGNTEAFF_TRBV12_TRBJ1-1, CASSYSDTGELFF_TRBV6_TRBJ2-2, CASSLTGNTEAFF_TRBV12_TRBJ1-1, CASSQGYEQYF_TRBV3_TRBJ2-7, CASSLSGNTIYF_TRBV12_TRBJ1-3. Among these five sequences, further extraction using the reject-by-exclusion method identifies CASSLSGNTIYF_TRBV12_TRBJ1-3 and CASSQGYEQYF_TRBV3_TRBJ2-7, while the remaining three sequences, CASSLGGNTEAFF_TRBV12_TRBJ1-1, CASSYSDTGELFF_TRBV6_TRBJ2-2, and CASSLTGNTEAFF_TRBV12_TRBJ1-1, contribute to discriminating the prevalence of glioma. With these three core sequences, high AUC and low correlation degree can be obtained in the Fig. 4E,G.

Using the same method, we analyze the dataset with a threshold of 0.001, which consists of a combination of 12 sequences. As is shown in Fig. 4D, the feature extraction algorithm employed is Adaboost, and the classification algorithm used is CV (CalibratedClassifierCV). This analysis also yields five sequences: CASSLGETQYF_TRBV12_TRBJ2-5, CASSLGGNQPQHF_TRBV12_TRBJ1-5, CASSLSGNTIYF_TRBV12_TRBJ1-3, CASSLQETQYF_TRBV12_TRBJ2-5, CASSLRETQYF_TRBV12_TRBJ2-5. With these five sequences, we achieve an AUC of 0.967 and accuracy, with a sensitivity of 0.933 and specificity of 1. Similarly, by screening one by one, we identify a combination of three sequences that also ensure high AUC values: CASSLGETQYF_TRBV12_TRBJ2-5, CASSLGGNQPQHF_TRBV12_TRBJ1-5, and CASSLSGNTIYF_TRBV12_TRBJ1-3. Meanwhile, Fig. 4F,H demonstrate that the combination of these three less correlated sequences can classify and achieve high classification AUC values. Lastly and not least, Fig. 4I is the seqlogo picture of the core three sequences at cutoff 1 data. Lasso and RFECV are used to extract features, and then several classification algorithms are used to classify the extracted sequence data. The corresponding classification results are shown in Supplementary Tables 3 and 4 respectively.

## Discussion

### Analysis of the results of the experiments

As we can see from the classification system constructed based on the diversity indices and the corresponding phenotype of the patients in Fig. 1, especially when the classification algorithm is Random Forest, the classification effect is really remarkable, and it can reach 100% differentiation. However, when we consider that the immune system of patients with major diseases is very different from that of healthy people, the AUC value of 1 is meaningless. A large difference in the diversity index can only indicate that the subject may be suffering from a certain disease, but it cannot pinpoint exactly what disease there is. Therefore, this is the significance of the need to target specific TCR sequences associated with gliomas.

By the principle of Fisher's exact test that the lower the P-value the stronger the correlation, we can roughly filter out the sequences with strong correlation with gliomas. The number of kinds of related sequences at different thresholds reveals a strong correlation with the glioma phenotype in the Fig. 2.

In addition, the multidimensional classification results of Fig. 3 can also enable it to show us that the 12 relevant sequences screened by Fisher's exact test are sufficient for the diagnosis of gliomas and can achieve a high classification accuracy. These 12 sequences are CASSLGGNQPQHF_TRBV12_TRBJ1-5, CASSLRETQYF_TRBV12_TRBJ2-5, CASLSGNTIYF_TRBV12_TRBJ1-3, CASLTGNTEAFF_TRBV12_TRBJ1-1, CASSFSGNTIYF_TRBV12_TRBJ1-3, CASSLGYEQYF_TRBV12_TRBJ2-7, CASFGGNTEAFF_TRBV12_TRBJ1-1, CASQGYEQYF_TRBV3_TRBJ2-7, CASLGETQYF_TRBV12_TRBJ2-5, CASSLQETQYF_TRBV12_TRBJ2-5, CASLGGGYTF_TRBV12_TRBJ1-2, CASLGGNTEAFF_TRBV12_TRBJ1-1. This result proves the validity of the commonly used Fisher's exact test.

However, because the thresholds to be set by the method are continuous, an infinite number of thresholds can be selected. For example, there are 12 sequences corresponding to a threshold value of 10^−3^, while there is only 1 sequence corresponding to a threshold value of 10^−4^. The exact number of sequences to which it is optimal to downsize to and the specific threshold between the two thresholds cannot be decided right away, and thus the method still has some limitations. The results indicate that achieving an AUC of 1 is more easily attainable in two-dimensional classification compared to multi-dimensional classification. However, the overall difference between the two approaches is not significant.

There are 2 comparative study to prove efficiency of proposed model. First, RFECV with Fisher’s exact test in Fig. 4 performances better than only Fisher’s exact test as feature selection measure in Fig. 3. In Fig. 3, we can only obtain the number of associated TCR sequences as 602, 39, 12, 1 corresponding to data’s cutoff at 0.1, 10^−2^, 10^−3^ and 10^−4^ respectively. When the cutoff is 10^−4^, the best accuracy and AUC are 0.767, and 10^−3^ is 0.967. But when we use RFECV and Fisher exact test together, 3 core TCR sequences were selected so that the best accuracy and AUC 0.967 can attain. Thus, using the fusion of RFECV and Fisher’s exact test can achieve better feature selection performance. Second, RFECV performances better than Lasso in the Core features’ selection of TCR sequences. Supplementary Tables 3 and 4 are the feature extraction method Lasso and the RFECV algorithm combined with different machine learning algorithms respectively. It can be seen that the best result of AUC and accuracy is 0.967, but the best dimension of lasso is 12, and the best dimension of RFECV combined with svm algorithm is 6. Therefore, the overall feature extraction effect of lasso is not as good as that of RFECV.

The results clearly demonstrate that for glioma diagnosis, higher accuracy can be achieved by utilizing data on the presence of multiple sets of 3D TCR β sequences. Regardless of the specific combination of sequences within each group, all combinations yield an AUC and accuracy of 0.967, with a sensitivity of 0.933 and specificity of 1. The first combination consists of CASSLGNTEAFF_TRBV12_TRBJ1-1, CASSLTGNTEAFF_TRBV12_TRBJ1-1, and CASSYSDTGELFF_TRBV6_TRBJ2-2. The second combination includes CASSLGETQYF_TRBV12_TRBJ2-5, CASSLGGNQPQHF_TRBV12_TRBJ1-5, and CASSLSGNTIYF_TRBV12_TRBJ1-3.

In summary, this study analyzes high-throughput sequencing data from glioma patients and healthy individuals. We explore multidimensional classification and feature selection of TCR sequence diversity indices, as well as two-dimensional classification and feature selection analysis of TCR-related sequences. As a result, we identify two sets of core sequences from these analyses. These two sets, each comprising three sequences, are sufficient to achieve a diagnostic accuracy of 96.7% in glioma detection, as evidenced by the AUC values. However, the reproducibility of these two sets of sequences in glioma patient data from different experimental contexts remains to be further investigated and validated.

Our research has demonstrated the practicality of this methodology not only for glioma diagnosis but also for the diagnosis of other viruses such as CMV and SARS-CoV-2, as well as various types of cancers. This methodology holds great promise for future cancer diagnosis, and we encourage other scientists to apply this approach to the diagnosis of different diseases. Our future work will focus on expanding the application of this methodology to further advance disease diagnosis.

On the whole, even though the experimental research in this paper is relatively comprehensive, there are still some limitations. For example, due to the limitations of experimental conditions, we can't test the effectiveness of the biomarkers we have obtained in clinical trials, and we can only verify them in the existing two sets of data with extremely high accuracy. If the following experimental conditions are met, it can be further verified and used for the development of immunotherapy or drugs such as TCR-T (engineered T cell receptor-T cell). In addition, the conclusion of this paper is still the high efficiency of diagnosis on a single cancer species or immune-related diseases, and future research can try whether this methodology has the same stable and high-precision diagnosis effect on the diagnosis of multiple immune diseases at the same time, such as early screening of multiple cancer species at the same time.

### Innovative analysis

To sum up, this paper is highly innovative. First of all, it is innovative to use TCR sequence as a marker. At present, most of the diagnosis of glioma is based on imaging, and the detection of body fluids is relatively few, and the study of TCR sequence as a biomarker is even less. Therefore, it is a great innovation to use TCRβ as a marker of glioma in this paper^19,20^; Secondly, high-throughput sequencing is a relatively rare and advanced biological information sequencing method, which can obtain the species information of millions or even hundreds of millions of dimensional sequences and creatively analyze extremely tiny biomarkers. However, the existing research on glioma diagnosis combined with high-throughput sequencing mainly studies the statistical differences between glioma and healthy people^11^; Accordingly, this paper is the first research on the diagnosis of glioma by combining artificial intelligence with high-throughput sequencing, which is extremely groundbreaking; Fourthly, we classified the high-throughput sequencing result data of three groups of different types of glioma by more than a dozen artificial intelligence algorithms, and get a relatively comprehensive classification method system of glioma and high-throughput sequencing. Finally, this paper is one of the few papers to extract the secondary features of related sequences. Firstly, Fisher exact test is used to traverse the P value according to the threshold of exponential change, and then RFECV, Lasso, and tree model, excluding a few sequences’ sets one by one and other feature selection methods are used for secondary extraction, and finally, the core related sequences are obtained.

## Methods

### Machine learning algorithms in different datasets

**Figure 5:**
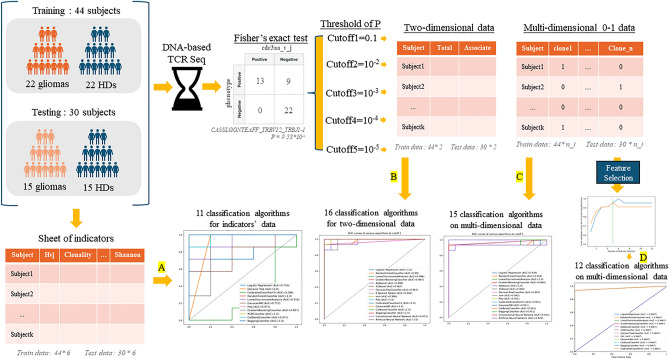
(**A**) represents bringing six diversity indexes of TCRβs into 11 classification algorithms, (**B**) represents bringing the two-dimensional data frame of TCR sequence into 16 classification algorithms, (**C**) represents bringing the multi-dimensional 0–1 data frame of TCRβs into 15 classification algorithms, and (**D**) represents bringing the multi-dimensional 0–1 data of TCRβs into 12 classification algorithms after feature selection process.

Figure 5 is a brief introduction to the experimental process of the whole paper. Firstly, two groups of glioma samples were obtained from two different experiments, and a corresponding number of HDs (healthy donors) were selected to form the training and testing data of this experiment, and the DNA-based TCR high-throughput sequencing was performed on them respectively. Fisher’s exact test was carried out on the sequenced data, and the P values corresponding to the prevalence of glioma in different sequences were obtained. According to the selection of the thresholds of different exponential levels of P value, the corresponding correlated sequence sets are obtained, and through these sequence sets, three different types of data structures are obtained and brought into the classification algorithms, which were divided into four experiments, representing at Fig. 5A–D.

### Feature selection algorithms

In the indices’ feature selection experiment, Tree-based algorithms such as RF, GBDT, XGBoost and permutation are applied to perform the selection. In the selection of core TCR sequences, this study primarily utilized two algorithms for feature extraction: Lasso and RFECV.

Tree-based algorithms such as Random Forest fall under the embedded methods of feature selection, making them suitable for feature extraction tasks. When dealing with high-dimensional data, feature selection becomes necessary, and it is a key approach to dimensionality reduction. Feature selection mainly consists of wrapper methods, filter methods, and embedded methods. Wrapper methods select features based on model performance by training a classifier on the selected feature set, examples include Recursive Feature Elimination (RFE), forward selection, and backward elimination. Filter methods, such as variance threshold, correlation coefficient, chi-square test, and Fisher exact test, select features based on statistical properties, screening features by setting thresholds or determining the number of features to select before training the model. Embedded methods automatically perform feature selection during the training process. The most typical examples are LASSO and decision tree algorithms, including Random Forest, XGBoost, and other tree models, which are frequently used in various studies^21^.

Feature selection is a crucial step in building efficient and accurate machine learning models, especially when dealing with high-dimensional datasets. In the feature selection process of TCR diversity indices’ data, we employed permutation importance combined with a Random Forest classifier to identify and rank the importance of features. We calculated the permutation importance for each feature. This method involves shuffling the values of each feature and measuring the decrease in model accuracy. Features that cause a significant drop in accuracy when shuffled are considered more important. By employing permutation importance in conjunction with a Random Forest classifier, we were able to effectively rank the features in our dataset. This approach not only enhances model interpretability but also aids in the feature selection process, ensuring that the final model is both efficient and accurate. This method is particularly useful in high-dimensional datasets where feature selection can significantly impact model performance^22^.

Lasso (Least Absolute Shrinkage and Selection Operator) is a regularization method commonly used for linear regression. It can be applied for both feature extraction, which involves generating new features from the original data, and feature selection, which involves identifying important features to retain. Lasso achieves feature selection by introducing an L1 regularization term to the loss function, allowing it to reduce the coefficients of unimportant features to zero. Unlike traditional methods, Lasso considers the collective effect of all features rather than evaluating the importance of each feature individually.

RFECV (Recursive Feature Elimination with Cross-Validation) is another feature extraction method that incorporates cross-validation. It recursively reduces the number of features and selects the optimal feature subset through cross-validation. Starting with all features, RFECV trains a model at each iteration and eliminates the least important features. Cross-validation is then utilized to evaluate the performance of each feature subset, ultimately selecting the optimal feature subset. RFECV determines the least important features based on model-based feature importance and eliminates them in each iteration. By repeatedly iterating, RFECV identifies the optimal feature subset for feature selection and extraction. One advantage of RFECV is its automatic selection of the number of features and utilization of cross-validation to choose the best feature subset, avoiding the subjectivity and limitations associated with manual feature selection. Additionally, the results obtained from RFECV can enhance the generalization ability and prediction performance of the model.

### Supplementary Information

Supplementary Information.

## Data Availability

The DNA-based TCR high-throughput sequencing data utilized in this study were obtained from multiple sources, including publicly available datasets published by *A vaccine targeting mutant IDH1 in newly diagnosed glioma*, *TCR Sequencing Can Identify and Track Glioma-Infiltrating T Cells after DC Vaccination* and the Adaptive Biotechnologies immuneACCESS, 10.21417/B7001Z. Detailed information on how to access and cite each specific dataset can be found in the respective references provided in this paper.

## Code availability

The original data and python code for the experiment are publicly available at https://github.com/Sarakyzhou/Glioma_classify_system/tree/main.
