# Supplementary material for: Comprehensive application of AI algorithms with TCR NGS data for glioma diagnosis
Source: Sci Rep. 2024 Jul 4;14:15361. doi: 10.1038/s41598-024-65305-9 (PMC11224284; doi:10.1038/s41598-024-65305-9)
Supplement: Supplementary file 1 — Supplementary Information. [file 41598_2024_65305_MOESM1_ESM.docx]

**Comprehensive application of AI algorithms with TCR NGS data for Glioma diagnosis**

Kaiyue Zhou^1^, Zhengliang Xiao^1^, Qi Liu^1^, Xu Wang^1^, Jiaxin Huo^1^, Xiaoqi Wu^1^, Xiaoxiao Zhao^1^, Xiaohan Feng^1^, Baoyi Fu^1^, Pengfei Xu^2^, Yunyun Deng^2^, Wenwen Xiao^2^, Tao Sun^2-3*^, Lin Da^1*^

**Authors’ affiliation:**

1 Department of Mathematics, School of Mathematical Sciences, Inner Mongolia University, Hohhot, China

2 Hangzhou ImmuQuad Biotechnologies, Hangzhou, China

3 Institute of Wenzhou, Zhejiang University, Wenzhou, China

* Corresponding Authors:

Tao Sun, PhD

Hangzhou ImmuQuad Biotech, LLC., Hangzhou, China

Institute of Wenzhou, Zhejiang University, Wenzhou, China

Telephone: 86-15982358665

1. mail: taosun@immuquad.com

Lin Da, PhD

Department of Mathematics, School of Mathematical Sciences, Inner Mongolia University, Hohhot, China

Telephone: 86-15647156090

E-mail: 111977331@imu.edu.cn

**Keywords:** glioma, T-cell repertoire (TCR), prognosis, Artificial Intelligence Techniques, feature selection methods

**Additional File:**

There is one Supplementary Material file available for this article.

**Acknowledgement and funding**

This work was supported by the Fundamental Research Funds for the Inner Mongolia University (21100-5187051).

**Abbreviations**

**LR:** Logistic Regression

**SVM:** Support Vector Machine

**LDA:** Linear Discriminant Analysis

**RF:** Random Forest

**NB:** Naive Bayes

**CBC:** CatBoost Classifier

**KNN:** K-Nearest Neighbors

**CV:** Calibrated Classifier

**POLY:** Polynomial SVM

**ANN:** Artificial Neural Network

**ADB:** AdaBoost

**CNN:** Convolutional Neural Network

**GBDT:** Gradient Boosting Decision Trees

**XGB:** Extreme Gradient Boosting (XGBoost)

**BAG:** Bagging Classifier

**DT:** Decision Tree

**TCR:** T-cell receptors

**CMV:** Cytomegalovirus

**CDR3:** Complementary Determining Region 3

**D-J:** beta chain

**V-J gene:** alpha chain

**TCRβs:** T-cell receptors beta chain

**AUC:** Area Under Curve

**Availability of data and materials**

The data that support the findings of this study are available from the Adaptive Biotechnologies immuneACCESS, https://doi.org/10.21417/B7001Z

**Declarations**

**Competing interests**

The authors declare no competing financial interests.

**Consent for publication**

All authors have approved the manuscript for submission

**Authors’ contributions**

LD and TS designed the study and revised the manuscript. KZ performed most of the experiments. KZ, YD, WX and PX processed the primary data. KZ, ZX, QL, XW, JH and XW built the ML algorithms for the study, KZ drafted the manuscript. KZ, XW, XZ, FX, BF helped to revise the manuscript. All authors checked and approved the final manuscript.

**Authors’ information**

Kaiyue Zhou, Zhengliang Xiao, Qi Liu, Xu Wang, Jiaxin Huo, Xiaoqi Wu, Xiaoxiao Zhao, Xiaohan Feng, Baoyi Fu & Lin Da

Department of Mathematics, School of Mathematical Sciences, Inner Mongolia University, Hohhot, China

Pengfei Xu, Yunyun Deng, Wenwen Xiao & Tao Sun

Hangzhou ImmuQuad Biotechnologies, Hangzhou, China

Tao Sun

Institute of Wenzhou, Zhejiang University, Wenzhou, China

1. **Algorithm and Training Details of the Article**

**A.1 Classification algorithms on TCR Diversity Indices**

We employed a comprehensive approach to train and fine-tune 11 different classification algorithms, ensuring each model's performance was optimized through a rigorous hyper-parameter search. This process involved training on a substantial dataset with consistent methodologies to allow for fair comparison across models. For Logistic Regression, we used the multinomial logistic regression variant with the `newton-cg` solver, balancing class weights and allowing a maximum of 10,000 iterations. This setup ensured robust handling of multiclass classification tasks. In contrast, Random Forest models were optimized by setting the number of estimators to 210, balancing the need for a diverse ensemble with computational efficiency. In the case of Support Vector Machine (SVM) with Polynomial Kernel, we utilized a polynomial degree of 3 and a regularization parameter (C) set to 1.0. This configuration was selected to capture non-linear relationships in the data effectively. Linear Discriminant Analysis (LDA) and K-Nearest Neighbors (KNN) models were employed with their default parameters, providing baselines for linear separability and local instance-based learning, respectively. For Naive Bayes (GaussianNB), we leveraged the model's default parameters, taking advantage of its simplicity and effectiveness in probabilistic classification. Gradient Boosting Decision Trees (GBDT) and XGBoost classifiers were also tuned using default parameters, ensuring powerful gradient-boosted ensemble learning techniques were employed. Decision Trees were used with their default settings, serving as interpretable models for understanding decision boundaries. Additionally, Calibrated Classifier with Naive Bayes incorporated a sigmoid method with cross-validation (cv=2) to adjust probabilistic outputs, enhancing reliability in probability estimates. CatBoost classifiers were fine-tuned with 5 iterations and a learning rate of 0.1, optimizing their gradient boosting capabilities on categorical features. Lastly, Bagging Classifier combined decision trees (with a base estimator of Decision Tree Classifier and random state of 1) through an ensemble of 50 trees, balancing variance reduction with model diversity.

**A.2 Binary classification algorithms on TCR sequences**

In the two-dimensional classification project on TCR sequences, we employed a comprehensive approach to train and fine-tune 16 different classification algorithms, ensuring each model's performance was optimized through a rigorous hyper-parameter search. This process involved training on a substantial dataset with consistent methodologies to allow for fair comparison across models. For Logistic Regression, we used the multinomial logistic regression variant with the `newton-cg` solver, balancing class weights and allowing a maximum of 10,000 iterations. This setup ensured robust handling of multiclass classification tasks. For the Support Vector Machine (SVM) with a linear kernel, we used a linear kernel with probability estimation enabled and a random state set to 10, optimizing for linear separability. The SVM with a polynomial kernel was configured with a polynomial degree of 3 and a regularization parameter (C) set to 1.0, effectively capturing non-linear relationships in the data.

The K-Nearest Neighbors (KNN) algorithm was optimized with the number of neighbors set to 10, balancing the trade-off between bias and variance. Random Forest models were optimized by setting the number of estimators to 210, balancing the need for a diverse ensemble with computational efficiency. Linear Discriminant Analysis (LDA) was employed with default parameters, providing a baseline for linear separability. The Gradient Boosting Decision Trees (GBDT) and XGBoost classifiers were also tuned using default parameters, ensuring powerful gradient-boosted ensemble learning techniques were employed.

AdaBoost was configured with 100 estimators, providing an effective boosting approach. The Decision Tree classifier used default settings, serving as an interpretable model for understanding decision boundaries. Naive Bayes (GaussianNB) leveraged the model's default parameters, taking advantage of its simplicity and effectiveness in probabilistic classification. For Calibrated Classifier with Naive Bayes, a sigmoid method with cross-validation (cv=2) was used to adjust probabilistic outputs, enhancing reliability in probability estimates.

CatBoost classifiers were fine-tuned with 5 iterations and a learning rate of 0.1, optimizing their gradient boosting capabilities on categorical features. The Bagging Classifier combined decision trees (with a base estimator of DecisionTreeClassifier and a random state of 1) through an ensemble of 50 trees, balancing variance reduction with model diversity.

For convolutional neural networks (CNN), we built a model with 32 filters in the Conv1D layer and used the Adam optimizer with a categorical cross-entropy loss function, training over 100 epochs with a batch size of 32. The artificial neural network (ANN) was structured with layers of 500, 500, 250, and 250 units respectively, all using the ReLU activation function, and a final softmax layer with 2 units for binary classification. The model was also trained using the Adam optimizer with a categorical cross-entropy loss function over 100 epochs and a batch size of 32. Both neural network models were evaluated on the test data using metrics such as accuracy, AUC, precision, and recall to ensure a comprehensive assessment of their performance.

**A.3 Multidimensional classification algorithms on TCR sequences**

In this project, we employed a comprehensive approach to train and fine-tune 15 different classification algorithms, ensuring each model's performance was optimized through a rigorous hyper-parameter search. This process involved training on a substantial dataset with consistent methodologies to allow for fair comparison across models. For Logistic Regression, we used the multinomial logistic regression variant with the `newton-cg` solver, balancing class weights and allowing a maximum of 10,000 iterations. This setup ensured robust handling of multiclass classification tasks. For the Support Vector Machine (SVM) with a linear kernel, we used a linear kernel with probability estimation enabled and a random state set to 10, optimizing for linear separability. The SVM with a polynomial kernel was configured with a polynomial degree of 3 and a regularization parameter (C) set to 1.0, effectively capturing non-linear relationships in the data. Random Forest models were optimized by setting the number of estimators to 210, balancing the need for a diverse ensemble with computational efficiency. Linear Discriminant Analysis (LDA) was employed with default parameters, providing a baseline for linear separability. The Gradient Boosting Decision Trees (GBDT) and XGBoost classifiers were also tuned using default parameters, ensuring powerful gradient-boosted ensemble learning techniques were employed.

AdaBoost was configured with 100 estimators, providing an effective boosting approach. The Decision Tree classifier used default settings, serving as an interpretable model for understanding decision boundaries. Naive Bayes (GaussianNB) leveraged the model's default parameters, taking advantage of its simplicity and effectiveness in probabilistic classification. For Calibrated Classifier with Naive Bayes, a sigmoid method with cross-validation (cv=2) was used to adjust probabilistic outputs, enhancing reliability in probability estimates.

CatBoost classifiers were fine-tuned with 5 iterations and a learning rate of 0.1, optimizing their gradient boosting capabilities on categorical features. The Bagging Classifier combined decision trees (with a base estimator of DecisionTreeClassifier and a random state of 1) through an ensemble of 50 trees, balancing variance reduction with model diversity.

For convolutional neural networks (CNN), we built a model with 32 filters in the Conv1D layer and used the Adam optimizer with a categorical cross-entropy loss function, training over 100 epochs with a batch size of 32. The artificial neural network (ANN) was structured with layers of 500, 500, 250, and 250 units respectively, all using the ReLU activation function, and a final softmax layer with 2 units for binary classification. The model was also trained using the Adam optimizer with a categorical cross-entropy loss function over 100 epochs and a batch size of 32. Both neural network models were evaluated on the test data using metrics such as accuracy, AUC, precision, and recall to ensure a comprehensive assessment of their performance.

**A.4 Feature Selection and Core features’ extraction on TCR sequences**

In the sequences’ feature selection process, feature extraction was performed using the RFECV algorithm in conjunction with nine different algorithms, followed by classification using twelve algorithms. The feature extraction process utilized AdaBoost (Adb), CatBoost (cbc), Decision Tree (dt), Gradient Boosting Decision Trees (gbdt), Linear Discriminant Analysis (lda), Logistic Regression (lr), Random Forest (rf), Support Vector Machine (svm), and XGBoost (xgb). The classification was carried out using Logistic Regression (lr), Random Forest (rf), Linear Discriminant Analysis (lda), Gradient Boosting Decision Trees (gbdt), AdaBoost (adb), XGBoost (xgb), Decision Tree (dt), Support Vector Machine (svm), Naive Bayes (nb), CatBoost (cbc), Bagging Classifier (bag), and Calibrated Classifier (cv).

For Logistic Regression, we used the multinomial logistic regression variant with the `newton-cg` solver, balancing class weights and allowing a maximum of 10,000 iterations. The Support Vector Machine (SVM) with a linear kernel was configured with probability estimation enabled and a random state set to 10. For the polynomial kernel SVM, we utilized a polynomial degree of 3 and a regularization parameter (C) set to 1.0. K-Nearest Neighbors (KNN) was optimized with 10 neighbors. Random Forest models were optimized by setting the number of estimators to 210. Linear Discriminant Analysis (LDA) was employed with default parameters. The Gradient Boosting Decision Trees (GBDT) and XGBoost classifiers were tuned using default parameters.

AdaBoost was configured with 100 estimators. Decision Tree classifiers were used with default settings. Naive Bayes (GaussianNB) leveraged default parameters. For Calibrated Classifier with Naive Bayes, a sigmoid method with cross-validation (cv=2) was used to adjust probabilistic outputs. CatBoost classifiers were fine-tuned with 5 iterations and a learning rate of 0.1. The Bagging Classifier combined decision trees with a base estimator of DecisionTreeClassifier and a random state of 1 through an ensemble of 50 trees, balancing variance reduction with model diversity.

This rigorous approach ensured each model's performance was optimized through a consistent and fair comparison, leveraging hyper-parameter tuning and feature extraction to enhance the effectiveness of cancer diagnosis based on the relevant sequence features.

1. **Tables**

**B.1 Supplementary Table1. The best classification performance of all 16 classification algorithms in the Binary classification project of TCR sequences.** Supplementary table 1 is the optimal index results of 16 algorithms in the two-dimensional classification of related sequences. This table is sorted according to the priority of higher AUC, higher accuracy and smaller cross entropy loss, and the obtained results are shown in the following table. For example, the best algorithm is nb (Naive Bayes), with highest AUC, accuracy and lowest cross-entropy loss, with the threshold of accuracy at 0.5523, and so on.

| data | algorithm | best_ threshold | AUC | Accuracy | Sensitivity | Specificity | loss |
| --- | --- | --- | --- | --- | --- | --- | --- |
| cutoff2 | nb | 0.5523 | 1.0000 | 1.0000 | 1.0000 | 1.0000 | 0.0451 |
| cutoff3 | cbc | 0.4516 | 1.0000 | 1.0000 | 1.0000 | 1.0000 | 0.5254 |
| cutoff1 | knn | 0.8000 | 1.0000 | 0.9667 | 0.9333 | 1.0000 | 0.0847 |
| cutoff2 | cv | 0.8173 | 1.0000 | 0.9667 | 0.9333 | 1.0000 | 0.1330 |
| cutoff3 | lr | 0.1714 | 1.0000 | 0.9667 | 0.9333 | 1.0000 | 0.1397 |
| cutoff2 | svm | 0.7677 | 1.0000 | 0.9667 | 0.9333 | 1.0000 | 0.1636 |
| cutoff3 | poly | 0.4516 | 1.0000 | 0.9667 | 0.9333 | 1.0000 | 0.3061 |
| cutoff2 | ANN | 0.0900 | 1.0000 | 0.9333 | 0.8667 | 1.0000 | 0.1020 |
| cutoff2 | adb | 0.2297 | 0.9956 | 0.9667 | 0.9333 | 1.0000 | 0.1519 |
| cutoff2 | lda | 0.2297 | 0.9956 | 0.9667 | 0.9333 | 1.0000 | 0.1519 |
| cutoff2 | CNN | 0.4300 | 0.9911 | 0.9333 | 0.8667 | 1.0000 | 0.2413 |
| cutoff2 | rf | 0.2571 | 0.9800 | 0.9667 | 0.9333 | 1.0000 | 0.1711 |
| cutoff2 | gbdt | 0.0000 | 0.9667 | 0.9667 | 0.9333 | 1.0000 | 0.3577 |
| cutoff2 | xgb | 0.0000 | 0.9667 | 0.9667 | 0.9333 | 1.0000 | 0.3577 |
| cutoff2 | bag | 0.8173 | 0.9667 | 0.9667 | 0.9333 | 1.0000 | 1.1513 |
| cutoff2 | dt | 0.0000 | 0.9667 | 0.9667 | 0.9333 | 1.0000 | 1.1513 |

**B.2 Supplementary Table2. The best classification performance of all 15 classification algorithms in the Multidimensional classification project of TCR sequences.** Supplementary table 2 is the optimal index results of 15 algorithms in the multi-dimensional classification of related sequences. This table is sorted according to the priority of higher AUC, higher accuracy and smaller cross entropy loss, and the obtained results are shown in the following table as well. For example, the best algorithm is adb (Adaboost), with highest AUC, accuracy and lowest cross-entropy loss, with the threshold of accuracy at 0, and so on. The threshold equals to 0 means when the prediction score equals to 0, the classification result is negative, else if the score is greater than 0 and less than or equal to 1, the prediction classification result is 1.

| data | algorithm | best_ threshold | AUC | Accuracy | Sensitivity | Specificity | loss |
| --- | --- | --- | --- | --- | --- | --- | --- |
| cutoff1 | adb | 0.0000 | 1.0000 | 0.9667 | 0.9333 | 1.0000 | 0.9655 |
| cutoff1 | ANN | 0.0700 | 0.9444 | 0.9333 | 0.8667 | 1.0000 | 0.9286 |
| cutoff3 | bag | 0.3359 | 0.9667 | 0.9667 | 0.9333 | 1.0000 | 0.9655 |
| cutoff2 | cbc | 0.3112 | 0.9667 | 0.9667 | 0.9333 | 1.0000 | 0.9655 |
| cutoff1 | CNN | 0.2900 | 0.9333 | 0.9333 | 0.8667 | 1.0000 | 0.9286 |
| cutoff3 | cv | 0.3359 | 0.9667 | 0.9667 | 0.9333 | 1.0000 | 0.9655 |
| cutoff1 | dt | 0.0000 | 0.9667 | 0.9667 | 0.9333 | 1.0000 | 0.9655 |
| cutoff3 | gbdt | 0.0000 | 0.9667 | 0.9667 | 0.9333 | 1.0000 | 0.9655 |
| cutoff1 | lda | 0.0000 | 1.0000 | 0.9667 | 0.9333 | 1.0000 | 0.9655 |
| cutoff3 | lr | 0.0871 | 0.9667 | 0.9667 | 0.9333 | 1.0000 | 0.9655 |
| cutoff3 | nb | 0.9982 | 0.9667 | 0.9667 | 0.9333 | 1.0000 | 0.9655 |
| cutoff3 | poly | 0.2254 | 0.9711 | 0.9667 | 0.9333 | 1.0000 | 0.9655 |
| cutoff3 | rf | 0.0000 | 0.9667 | 0.9667 | 0.9333 | 1.0000 | 0.9655 |
| cutoff3 | svm | 0.0995 | 0.9667 | 0.9667 | 0.9333 | 1.0000 | 0.9655 |
| cutoff3 | xgb | 0.0000 | 0.9667 | 0.9667 | 0.9333 | 1.0000 | 0.9655 |

**B.3 Supplementary Table3. The best classification performance of all 13 algorithms with Lasso feature selection method in the TCR sequences’ Multidimensional classification project.** Supplementary table 3 is the optimal index results of 13 algorithms in the multi-dimensional classification of related sequences. This table is sorted like the above two table. As we can see from the table, when the feature selection method is Lasso and classification algorithm is Adboost, 11 sequences can attain the highest AUC and accuracy at 0.967.

| data | Classify_  method | dimension | best_ threshold | AUC | Accuracy | Sensitivity | Specificity | loss |
| --- | --- | --- | --- | --- | --- | --- | --- | --- |
| cutoff2 | adb | 11 | 0.0000 | 0.9667 | 0.9667 | 0.9333 | 1.0000 | 0.9655 |
| cutoff2 | bag | 11 | 0.2332 | 0.9667 | 0.9667 | 0.9333 | 1.0000 | 0.9655 |
| cutoff2 | cbc | 11 | 0.4192 | 0.9667 | 0.9667 | 0.9333 | 1.0000 | 0.9655 |
| cutoff2 | cv | 11 | 0.4192 | 0.9667 | 0.9667 | 0.9333 | 1.0000 | 0.9655 |
| cutoff2 | dt | 11 | 0.0000 | 0.9000 | 0.9000 | 0.8000 | 1.0000 | 0.8889 |
| cutoff3 | gbdt | 9 | 0.0000 | 0.9667 | 0.9667 | 0.9333 | 1.0000 | 0.9655 |
| cutoff2 | lda | 11 | 0.0000 | 0.9667 | 0.9667 | 0.9333 | 1.0000 | 0.9655 |
| cutoff2 | lr | 11 | 0.0882 | 0.9667 | 0.9667 | 0.9333 | 1.0000 | 0.9655 |
| cutoff2 | nb | 11 | 0.9954 | 0.9667 | 0.9667 | 0.9333 | 1.0000 | 0.9655 |
| cutoff2 | poly | 11 | 0.2332 | 0.9689 | 0.9667 | 0.9333 | 1.0000 | 0.9655 |
| cutoff2 | rf | 11 | 0.0000 | 0.9667 | 0.9667 | 0.9333 | 1.0000 | 0.9655 |
| cutoff1 | svm | 18 | 0.0890 | 0.9667 | 0.9667 | 0.9333 | 1.0000 | 0.9655 |
| cutoff3 | xgb | 9 | 0.0000 | 0.9667 | 0.9667 | 0.9333 | 1.0000 | 0.9655 |

**B.4 Supplementary Table4. The best classification performance of all 12 classification algorithms with RFECV feature selection method in the TCR sequences’ Multidimensional classification project.** In supplementary table 4, RFECV algorithm combines 9 machine learning algorithms to classify, and then uses 12 classification algorithms to classify the classification results, and obtains the best classification results corresponding to each classification algorithm. As we can see from the table, when the feature selection method is the combination of RFECV and svm, classification algorithm is svm, 6 sequences can attain the highest AUC and accuracy at 0.967.

| cutoff | Selection_  method | classify_  method | dimension | auc | acc | sens | spec | loss |
| --- | --- | --- | --- | --- | --- | --- | --- | --- |
| cutoff3 | svm | svm | 6 | 0.9667 | 0.9667 | 0.9333 | 1.0000 | 0.1445 |
| cutoff3 | lda | cv | 7 | 0.9667 | 0.9667 | 0.9333 | 1.0000 | 0.1629 |
| cutoff3 | xgb | lr | 6 | 0.9667 | 0.9667 | 0.9333 | 1.0000 | 0.1803 |
| cutoff3 | gbdt | dt | 7 | 0.9667 | 0.9667 | 0.9333 | 1.0000 | 1.1513 |
| cutoff3 | dt | nb | 7 | 0.9667 | 0.9667 | 0.9333 | 1.0000 | 1.1513 |
| cutoff3 | dt | rf | 6 | 0.9667 | 0.9667 | 0.9333 | 1.0000 | 1.1697 |
| cutoff3 | cbc | xgb | 6 | 0.9667 | 0.9333 | 0.8667 | 1.0000 | 0.2206 |
| cutoff3 | rf | cbc | 6 | 0.9667 | 0.9333 | 0.8667 | 1.0000 | 0.5613 |
| cutoff3 | lr | bag | 6 | 0.9667 | 0.9333 | 0.8667 | 1.0000 | 1.1893 |
| cutoff3 | adb | lda | 6 | 0.9667 | 0.9000 | 0.8000 | 1.0000 | 0.3417 |
| cutoff3 | lr | adb | 12 | 0.9667 | 0.9000 | 0.8000 | 1.0000 | 0.6529 |
| cutoff3 | gbdt | gbdt | 12 | 0.9667 | 0.9000 | 0.8000 | 1.0000 | 0.8356 |

1. **Additional Files**

N/A
